# Lost in the Translation Trap: Quest for a Research Reporting Culture That More Carefully Weighs Clinical Applicability in Experimental Disease Models

**DOI:** 10.3389/fncel.2021.800207

**Published:** 2021-11-26

**Authors:** Dirk M. Hermann

**Affiliations:** Department of Neurology, University Hospital Essen, University of Duisburg-Essen, Essen, Germany

**Keywords:** clinical relevance, human patient, therapy, animal model, clinical translation

Translating therapeutic findings from cellular systems or animals to human patients is a huge challenge. Clinical breakthroughs are rare, and the vast majority of research efforts are getting stuck on their way to clinical application (Endres et al., 2008; Banik et al., 2015). This roadblock is particularly eminent in neurosciences, which are devoted to an extremely complex tissue, the brain, which is surrounded by the well-regulated and tight blood-brain barrier, that prevents the brain access of a vast majority of pharmacological or cell-based therapeutics. In many disease areas, specifically in ischemic stroke or neurodegenerative diseases, we do not have any disease-modifying therapies at hand, allowing us to alleviate clinical consequences, once the disease has manifested its injury.

The scientific community and research funding organizations are well aware of the need of causatively acting therapies in several disease areas, prioritizing projects and papers potentially capable of facilitating clinical breakthroughs. The almost exponential increase of Pubmed listed papers in these disease areas underlines this fact (Figure 1). On the other hand, stringent research concepts with realistic chances of successful clinical translation are seldom. They usually require focused efforts of well-established groups over many years, performing thorough investigations in a variety of model systems, including animal models mimicking clinical risk factors and associated diseases, as well as investigations in different animal species and lab environments, before proof-of-concept studies in human patients can realistically be considered. These clinical studies then require well-defined study centers, in which disease responses in the animal are replicated in the human in a meaningful way.

**Figure 1:**
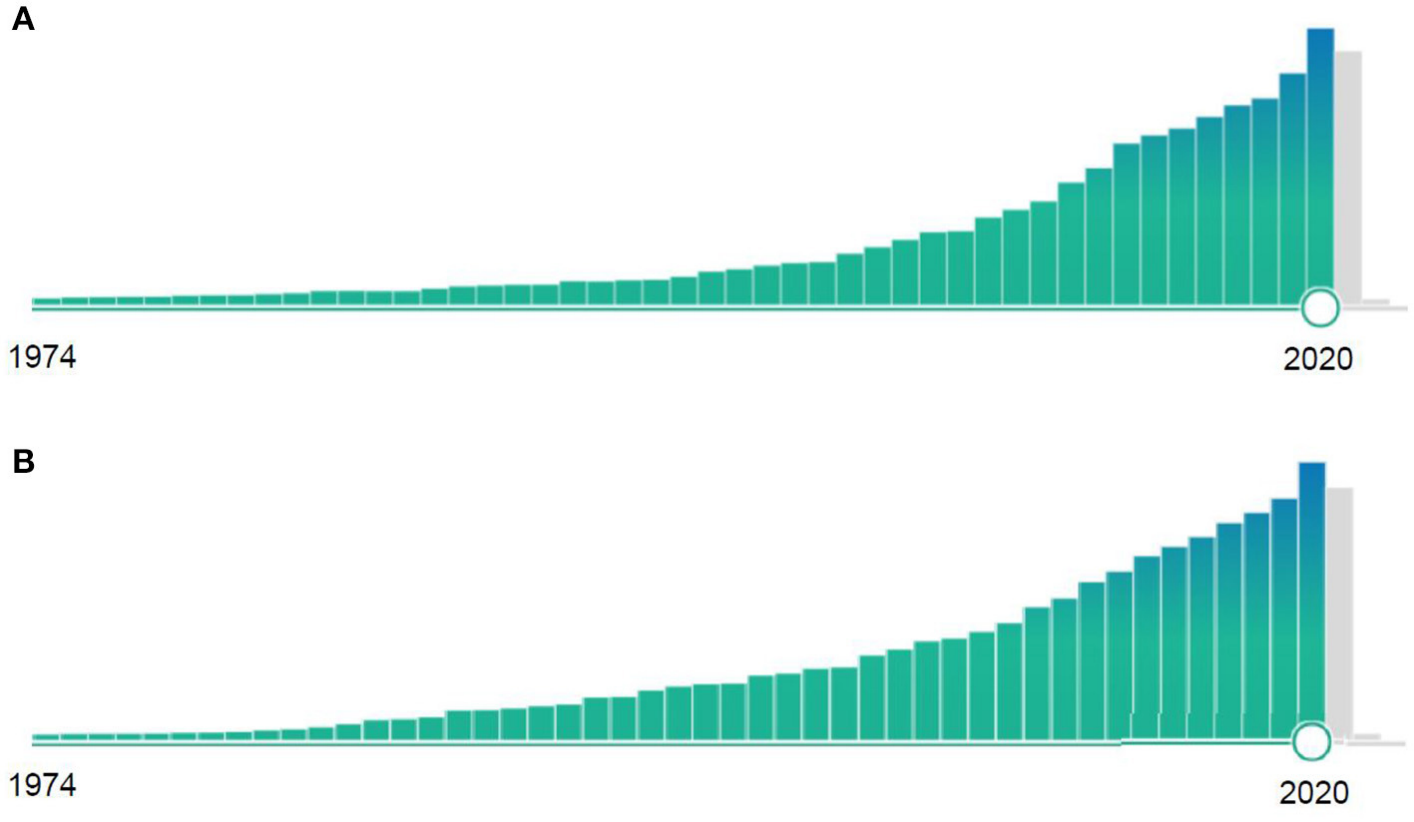
Relative number of Pubmed-listed publications containing the key words **(A)** “Ischemic stroke” or **(B)** “Alzheimer” from 1974 to 2020. Note the almost exponential increase of research publications during these 47 years.

In view of the enormous challenges of this clinical translation, there is an inflation of text paragraphs in scientific research papers, pointing out clinical relevancy of sometimes circumscribed research findings, for which this relevancy has not thoroughly been reflected. Introductions claiming that there is a need for new therapies in clinical conditions like stroke or Alzheimer's disease have little value for the scientific community, when theses paragraphs appear in almost stereotypical way in front of research papers. In most cases, the papers will not be able to provide silver linings for solutions of these needs. Likewise, conclusion paragraphs stating that the successful modulation of molecules by a given treatment in sometimes highly artificial disease systems may offer benefits for the clinical needs of patients do not have any added value to research papers unless supported by grounded concepts that indeed make their clinical translation promising. Such text paragraphs do not respect the complexity of efforts required in order to bring new treatments into clinical therapy, and the interventions presented are in many cases not seriously intended by the authors to be put forward to clinical proof-of-concept studies.

With this Specialty Grand Challenge I would like to quest for a new culture of more critical discussion of translational perspectives of research findings in scientific publications. I would like to ask all authors to more critically weigh statements on the potential clinical translation of their research findings and encourage reviewers, associate editors and chief editors to question groundless statements suggesting clinical applicability, unless substantiated by a critical mass of data, which deserve in depth discussion of their translational value in front of scientific audiences. We currently have a lack of an academic culture critically assessing clinical application perspectives, and a more thorough discussion may help to identify therapies, which are worth to be evaluated in the clinical setting. In this sense, self-containment may help to improve scientific quality at the transition from the laboratory bench to the patient bed.

## Author Contributions

DMH prepared the paper and finalized it.

## Conflict of Interest

The author declares that the research was conducted in the absence of any commercial or financial relationships that could be construed as a potential conflict of interest.

## Publisher's Note

All claims expressed in this article are solely those of the authors and do not necessarily represent those of their affiliated organizations, or those of the publisher, the editors and the reviewers. Any product that may be evaluated in this article, or claim that may be made by its manufacturer, is not guaranteed or endorsed by the publisher.
